# Taxonomic recovery of the ant cricket *Myrmecophilus albicinctus* from *M. americanus* (Orthoptera, Myrmecophilidae)

**DOI:** 10.3897/zookeys.589.7739

**Published:** 2016-05-16

**Authors:** Takashi Komatsu, Munetoshi Maruyama

**Affiliations:** 1 Kyushu University, Hakozaki 6-10-1, Higashi-ku, Fukuoka 812-8581 Fukuoka, Japan

**Keywords:** Formicidae, host specificity, myrmecophily, symbiont, synonymy

## Abstract

*Myrmecophilus americanus* and *Myrmecophilus albicinctus* are typical myrmecophilous insects living inside ant nests. These species are ecologically important due to the obligate association with tramp ant species, including harmful invasive ant species. However, the taxonomy of these “white-banded ant crickets” is quite confused owing to a scarcity of useful external morphological characteristics. Recently, *Myrmecophilus albicinctus* was synonymized with *Myrmecophilus americanus* regardless of the apparent host use difference. To clarify taxonomical relationship between *Myrmecophilus albicinctus* and *Myrmecophilus albicinctus*, we reexamined morphological characteristics of both species mainly in the viewpoint of anatomy. Observation of genitalia parts, together with a few external body parts, revealed that *Myrmecophilus albicinctus* showed different tendency from them of *Myrmecophilus americanus*. Therefore, we recover *Myrmecophilus albicinctus* as a distinct species on the basis of the morphology.

## Introduction

**Figure 1.**
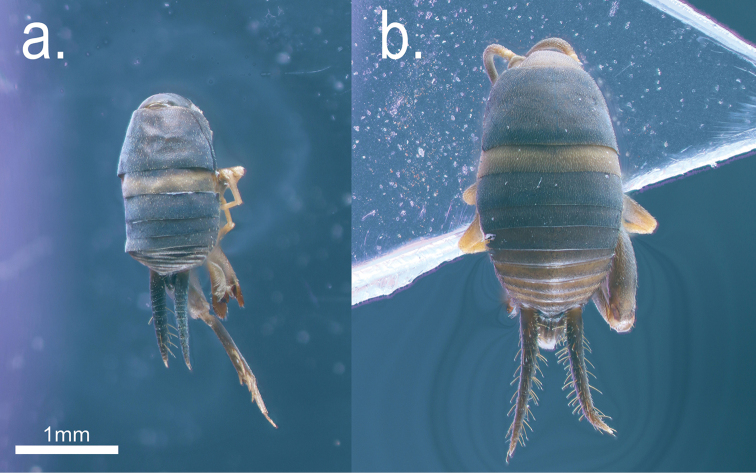
Specimens of the two *Myrmecophilus* species in dorsal view: *Myrmecophilus americanus* (**a**) and *Myrmecophilus albicinctus* (**b**).

**Figure 2.**
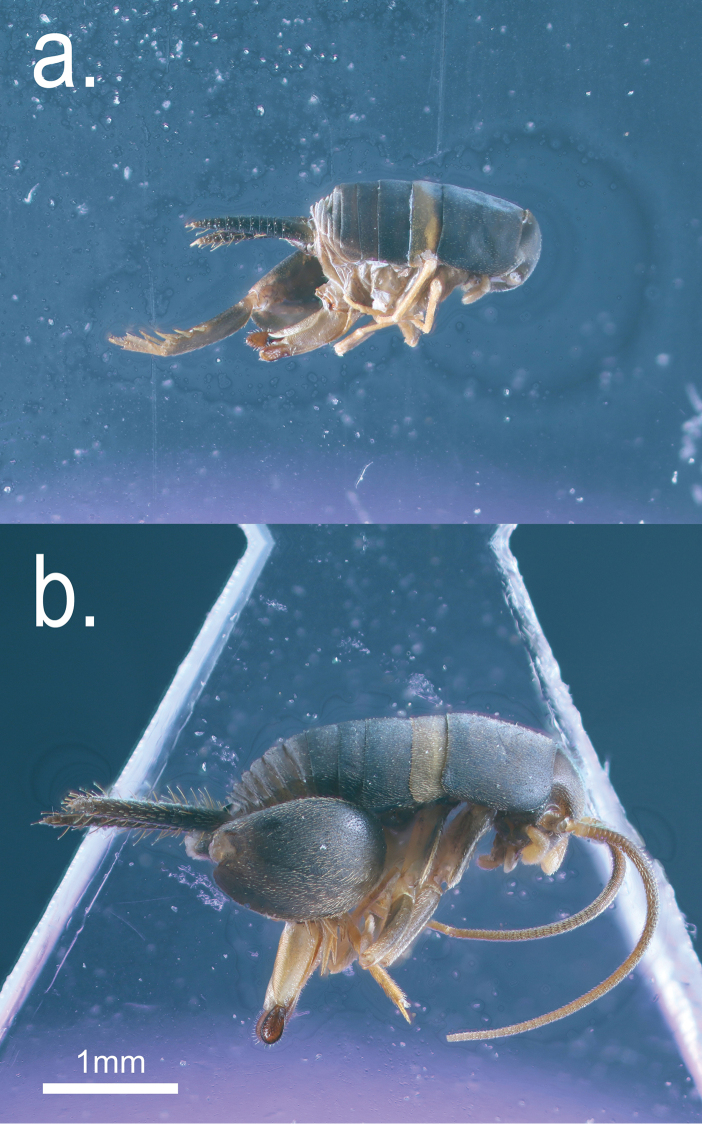
Specimens (same as those shown in Fig. 1) of the two *Myrmecophilus* species in lateral view: *Myrmecophilus americanus* (**a**) and *Myrmecophilus albicinctus* (**b**).

Myrmecophilus (Myrmophilina) americanus Saussure, 1877 (Orthoptera: Myrmecophilidae) (Figs 1a, 2a) is a typical example of an ant guest that lives inside ant nests. This species, similar to its congeners, eats food found inside the ant nest, either by itself or via mouth-to-mouth feeding by the ants (Wetterer and Hugel 2008). Its body color is totally black except for a single white band on the mesonotum. *Myrmecophilus americanus* was first described on the basis of a single female specimen collected in Colombia (Saussure 1877). The species is currently known to be distributed across tropical Asia, including on small islands, northern Africa, and the Neotropics (Wetterer and Hugel 2008). Its host-ant-species specificity is quite high; it has been collected exclusively (Wetterer and Hugel 2008) from nests of the longhorn crazy ant, *Paratrechina longicornis* (Latreille 1802). Because of its broad distribution, however, specimens of *Myrmecophilus americanus* from several localities have been given different species names. For example, Wasmann (1905) described *Myrmecophilus prenolepidis* from India, and Gorochov (1994) described Myrmecophilus (Eumyrmecophilus) microscopicus from Seychelles, but both these species have since been synonymized with *Myrmecophilus americanus*, the former by Schimmer (1909) and the latter by Hugel (2006). On the other hands, Ebner (1956) described *Myrmecophilus robustus* from Egypt though it has been also synonymized with *Myrmecophilus americanus* by Chopard (1968).

*Myrmecophilus albicinctus* (Chopard 1924) (Figs 1b, 2b) was first described on the basis of four females (the holotype and three paratypes) collected from India, but Ingrisch (2010) considered this species to be indistinguishable from *Myrmecophilus americanus* due to the similarity of their morphological characteristics. In contrast to *Myrmecophilus americanus*, however, *Myrmecophilus albicinctus* is known from only tropical Asia, including small islands (Maruyama 2006, Murai and Ito 2011). Moreover, recent studies have indicated that it is exclusively found in nests of the yellow crazy ant, *Anoplolepis gracilipes* (Maruyama 2006, Komatsu et al. 2009, Murai and Ito 2011), although the holotype specimen was collected from a *Camponotus mitis* nest (Chopard 1924). Laboratory experiments have shown that, from the perspective of behavioral ecology, *Myrmecophilus albicinctus* is closely dependent on *Anoplolepis gracilipes* (Komatsu et al. 2009). With regard to the taxonomy of this species, Ichikawa (2001), independently of Hugel and Blard (2005) and Hugel (2006), synonymized *Myrmecophilus microscopicus* with *Myrmecophilus albicinctus*. Thus, the taxonomic status of *Myrmecophilus americanus* and *Myrmecophilus albicinctus* is quite confused (Ingrisch 2010). The host ant species of both *Myrmecophilus americanus* and *Myrmecophilus albicinctus* are well-known tramp ants, and *Anoplolepis gracilipes* in particular is known to be a highly destructive invasive species. From the viewpoint of pest control, therefore, the taxonomy of parasites and myrmecophilous insects associated with this invasive ant species is of fundamental interest.

Recently, Ingrisch (2010) synonymized *Myrmecophilus albicinctus* with *Myrmecophilus americanus* because no morphological characteristic except body size was found to clearly distinguish them. However, we have previously suggested, following Wetterer and Hugel (2008), that each of these two species depends strictly on a different host ant species, and, moreover, we showed by a preliminary molecular phylogenetic analysis that they can be genetically differentiated (Komatsu et al. 2009, Komatsu et al. unpublished). In addition, we have found clear morphological differences between these two *Myrmecophilus* species.

For recovery of ”*Myrmecophilus albicinctus*”, there is problem of validity to use the name toward the species. As above mentioned, *Myrmecophilus albicinctus* have once synonymized with *Myrmecophilus americanus*. In addition, there is an older synonym of *Myrmecophilus americanus*; that is, *Myrmecophilus prenolepidis*. Under normal circumstances, it should be used the name of *Myrmecophilus prenolepidis* toward the recovered species. However, *Myrmecophilus prenolepidis* was described on the basis of specimens collected from nest of *Prenolepis longicornis* (Roger, 1863) that is synonymized as *Paratrechina longicornis* (Emery 1925). The host specificity of *Myrmecophilus albicinctus* toward *Anoplolepis gracilipes* is strong in principle so it is unlikely that it is collected from nests of *Paratrechina longicornis*. Given this, the specimens that formerly regards as *Myrmecophilus prenolepidis* can be regarded as not *Myrmecophilus albicinctus* but *Myrmecophilus americanus* which we call in present paper. Therefore, we apply the name of *Myrmecophilus albicinctus* for the recovered species. A series of old and recent host ant species records for *Myrmecophilus americanus* and *Myrmecophilus albicinctus* are listed in Table 1.

**Table 1.** Past literatures including host ant record of *Myrmecophilus americanus* and *Myrmecophilus albicinctus*. For records of *Myrmecophilus americanus*, Wetterer and Hugel (2008) have written up in detail.

Recorded species	Host ant species	Author
*Myrmecophilus prenolepidis*	*Prenolepis* (= *Paratrechina*) *longicornis*	Wasmann (1905)
*Myrmecophilus albicincta* (=*albicinctus*)	*Camponotus mitis*	Chopard (1924)
*Myrmecophilus robustus*	*Camponotus* sp.	Ebner (1956)
*Myrmecophilus albicinctus*	*Anoplolepis gracilipes*	Ichikawa et al. 2000
*Myrmecophilus microscopicus*	*Paratrechina longicornis*	Hugel and Blard (2005)
*Myrmecophilus albicinctus*	*Anoplolepis gracilipes*, *Pheidole* spp. (the latter is quite rare case)	Maruyama (2006)
*Myrmecophilus americanus*	*Paratrechina longicornis*, *Camponotus* sp. (the latter is only single record)	Wetterer and Hugel (2008)
*Myrmecophilus albicinctus*	*Anoplolepis gracilipes*	Komatsu et al. (2009)
*Myrmecophilus albicinctus*	*Anoplolepis gracilipes*	Murai and Ito (2011)
*Myrmecophilus americanus*	*Paratrechina longicornis*	Wetterer and Hugel 2014

## Methods

### Sampling

Field sampling of *Myrmecophilus americanus* and *Myrmecophilus albicinctus* in the Ryukyu Islands and in southeast Asia was conducted from 2005 to 2015. Ant crickets were collected from nests of *Anoplolepis gracilipes* and *Paratrechina longicornis* by locating nest entrances, turning over stones, or breaking up decayed logs and stumps. Whenever ant crickets were found, as many as possible were collected and preserved in absolute alcohol.

### Examination of samples

One of us (TK) examined specimens that he collected or were collected by colleagues. In addition, he visited the Muséum national d’Histoire naturelle (MNHN) to examine both type specimens (*Myrmecophilus americanus* and *Myrmecophilus albicinctus*).

The collected ethanol-preserved specimens were used for morphological observation. Specimens were dissected to observe their genitalia (abdominal terminalia). Each specimen was softened before dissection by warming (60 °C for 30–60 min) it in a small ceramic bowl (2.5 cm in diameter) with a small amount of water. Then, the specimen was dissected in water at high magnification under a stereomicroscope (Olympus SZ-40, ×6.7–80). The abdominal apex was removed from each specimen and dissected. Body parts were soaked in a warm 5–8% solution of potassium chloride (60 °C, 20–60 min), cleaned in 30% ethanol (5 min), and dehydrated in 99% ethanol (5 min). The dehydrated materials were mounted in Euparal (Chroma-Gesellschaft) on glass slides for detailed observation.

## Results

### Taxonomy

#### Myrmecophilus (Myrmophilina) americanus

Taxon classificationAnimaliaOrthopteraMyrmecophilidae

Saussure, 1877

##### Material examined.

3♂ and 1♀, collected from 50 Ngamwongwan Rd. ChatuChak Bangkok, Thailand, 6-X-2007, Komatsu T.; 1♀, Plot 256, Tingkat Perusahaan 5, Kawasan Perindustrian Perai 2, Perai, Penang, Malaysia, 28-I-2011, Sumino T.; 1♀, Andalas University, Jl. Limau Manis, Kecamatan Pauh, Padang, Sumatera Barat 25163, Indonesia, 27-XI-2013, Komatsu T.; 1♂, Lembaga Ilmu Pengetahuan Indonesia, JL. Raya Jakarta Bogor km 46, Cibinong 16911, Indonesia, 20 VI 2013, Komatsu T.

##### Type material.

**Figure 3.**
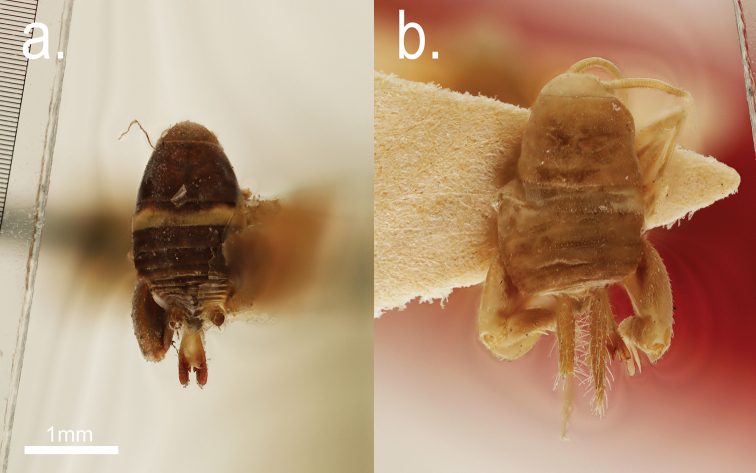
Type specimens: *Myrmecophilus americanus* (**a**) and *Myrmecophilus albicinctus* (**b**).

Syntype 1♀: Barkuda Id., Chilka Lake, Ganjam dist., Madras Pres. 4-19-1919. F. H. Gravery, Zool. Surv. Ind. (MNHN) (Fig. 3a)

##### Diagnosis.

**Figure 4.**
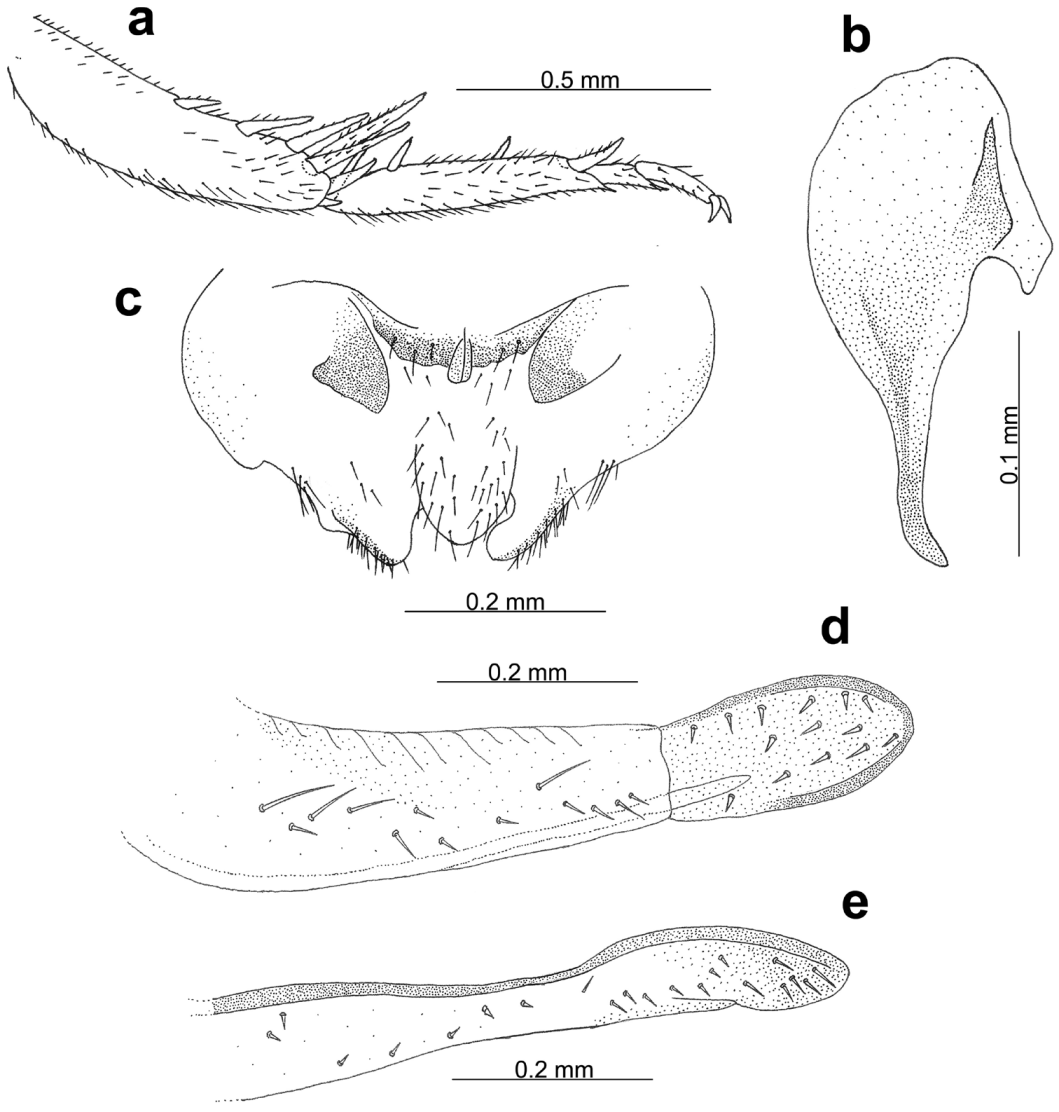
*Myrmecophilus americanus*. Hind tibia and tarsus, male inferior view (**a**); male pseudepiphallic ancora (**b**); male abdominal apex showing tubercles of the last abdominal tergite (**c**); female ovipositor, lateral view, dorsal margin (**d**); female ovipositor, lateral view, ventral margin (**e**).

Hind tarsus is relatively short (less than 1 mm, Fig. 4a); male phallic complex with pseudepiphallic ancorae short and roughly rounded with no dorsal branch. Ventral appendage of pseudepiphallic ancora somewhat predominant with both ends roughly square (Fig. 4b); male tenth abdominal tergite bituberculate, with scarce hair but without long strong spines (Fig. 4c); female ovipositor notably short and spoon-shaped in lateral view. Apical valves on both dorsal and ventral margins rounded, more than in other *Myrmecophilus* species (both *Myrmecophilus americanus* and *Myrmecophilus albicinctus* have rounded valves, with those of latter being more rounded) (Fig. 4d, e).

#### Myrmecophilus albicinctus

Taxon classificationAnimaliaOrthopteraMyrmecophilidae

Chopard, 1924 sp. rev.

##### Material examined.

1♀, collected from Koshidake, Iheya-jima, Okinawa, Japan, 5-IV-1996, Inada S.; 2♂, Gusukube-sunagawa, Miyakojima-shi, Miyako-jima, Okinawa, Japan, 8-VI-1996, Inada S.; 1♂ and 1♀, collected from Urasoe, Okinawa-jima, Okinawa, Japan, 24-VII-2007, Komatsu T.; 3♂ and 1♀, Yona, Kunigami-son, Okinawa-jima, Okinawa, Japan, 6-VIII-2007, Komatsu T.; 2♀, Field Studies Centre of the University of Malaya, Ulu Gombak, Selangor, Malaysia, 25-X-2012, Komatsu T.; 3♀, Bogor Botanical Gardens, Jalan Ir. Haji Juanda No.13, 16122, Indonesia, 22-XI-2013, Komatsu M.; 1♂, Andalas University, Jl. Limau Manis, Kecamatan Pauh, Padang, Sumatera Barat 25163, Indonesia, 1-XII-2013, Komatsu M; 1♂, 16 km Point, Kaeng Krachan National Park, Phetchaburi, Thailand, 28-VI-2014, Komatsu T.; 4♂ and 2♀, Jalan Universiti, 50603 Kuala Lumpur, Wilayah Persekutuan Kuala Lumpur, Malaysia, 28-XI-2005, Komatsu T.; 2♂ and 1♀, Daruma-yama, Kume-jima, Okinawa, Japan, 9-XII-2014, Komatsu T.

##### Type material.

Paratype 2♂2♀: Pattambi, Molabas Dist., F. H. Gravely V. 30 and *Anoplolepis longipes*. (Fig. 3b).

##### Diagnosis.

**Figure 5.**
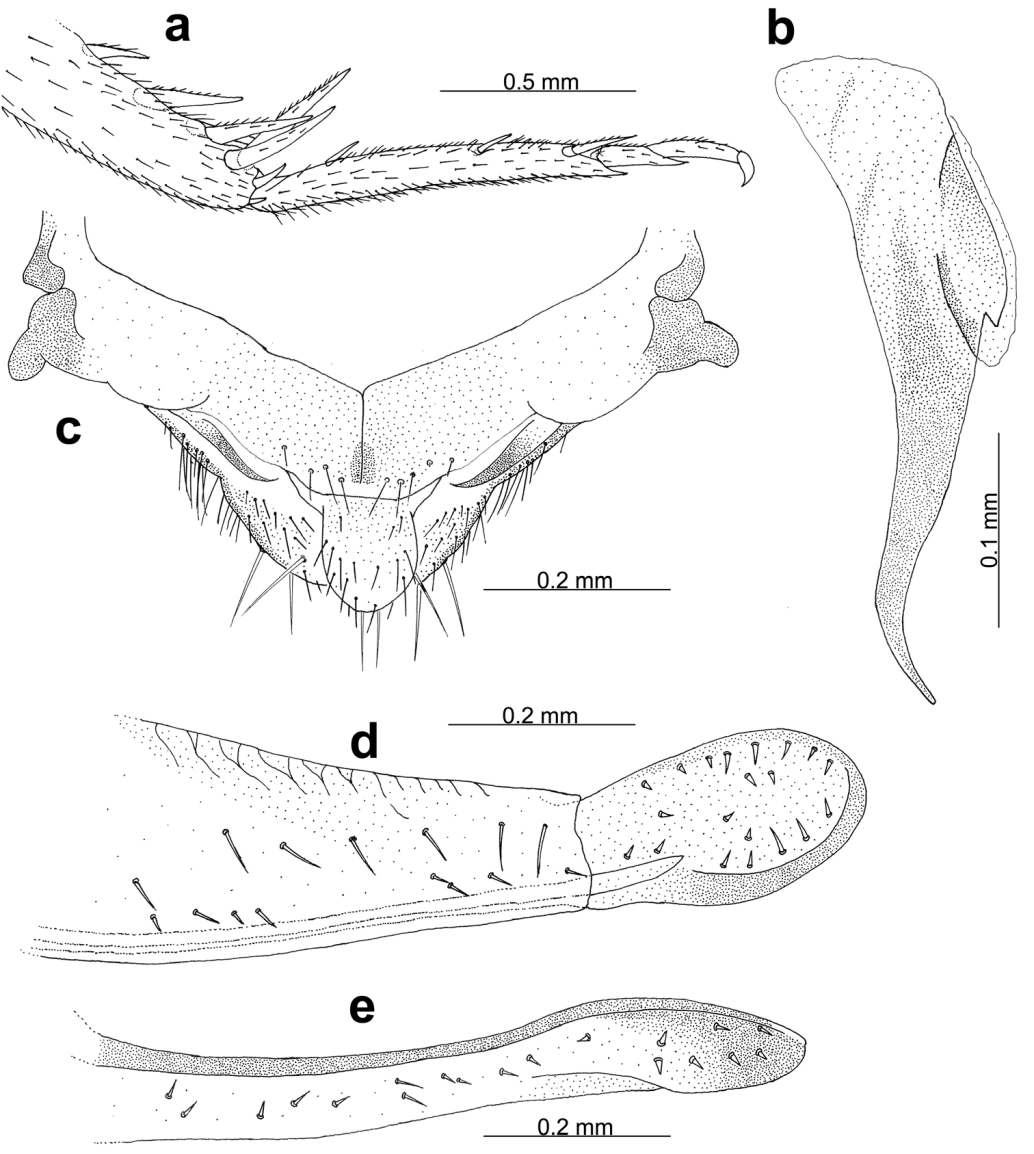
*Myrmecophilus albicinctus*. Hind tibia and tarsus, male inferior view (**a**); male pseudepiphallic ancora (**b**); male abdominal apex showing tubercles of the last abdominal tergite (**c**); female ovipositor, side view, dorsal margin (**d**); female ovipositor, side view, ventral margin (**e**).

Hind tarsus is relatively long (more than 1 mm, Fig. 5a); male phallic complex with pseudepiphallic ancorae straightly elongate with no dorsal branch. Ventral appendage of pseudepiphallic ancora considerably reduced with both ends angular (Fig. 5b); male tenth abdominal tergite bituberculate, with rich hair and long strong spines (Fig. 5c); female ovipositor closely resembles that of *Myrmecophilus americanus*, except the apical valve on the dorsal margin is more rounded in lateral view (Fig. 5d, e).

##### Remark.

This species can be clearly discriminated from *Myrmecophilus americanus* on the basis of the described diagnostic characteristics. Therefore, we recognize *Myrmecophilus albicinctus* as a distinct species.

## Discussion

With regard to the taxonomy of *Myrmecophilus* ant crickets, Ingrisch (2010) has stated that better characteristics than host specificity are needed to differentiate species. In fact, some species of *Myrmecophilus* are host-generalists and do not show any apparent host specificity (Komatsu et al. 2009) whereas other *Myrmecophilus* species, including *Myrmecophilus americanus* and *Myrmecophilus albicinctus*, are characterized by strict host-species specificity (Wetterer and Hugel 2008; Komatsu et al. 2009). It has been suggested that host-species differentiation is one cause of speciation (Schönrogge et al. 2002, Ugelvig et al. 2011). Given the similarities of *Myrmecophilus americanus* and *Myrmecophilus albicinctus*, they may represent a transitional phase of speciation via host switching.

## Supplementary Material

XML Treatment for Myrmecophilus (Myrmophilina) americanus

XML Treatment for Myrmecophilus albicinctus
